# Wearable Sensor-Based Real-Time Gait Detection: A Systematic Review

**DOI:** 10.3390/s21082727

**Published:** 2021-04-13

**Authors:** Hari Prasanth, Miroslav Caban, Urs Keller, Grégoire Courtine, Auke Ijspeert, Heike Vallery, Joachim von Zitzewitz

**Affiliations:** 1 ONWARD, Building 32, Hightech Campus, 5656 AE Eindhoven, The Netherlands; hari.prasanth@onwd.com; 2 Faculty of Mechanical, Maritime and Materials Engineering, Delft University of Technology, Mekelweg 2, 2628 CD Delft, The Netherlands; 3 Institute of Bioengineering, École Polytechnique Fédérale de Lausanne (EPFL), 1015 Lausanne, Switzerland; miroslav.caban@epfl.ch (M.C.); auke.ijspeert@epfl.ch (A.I.); 4 ONWARD, EPFL Innovation Park Building C, 1015 Lausanne, Switzerland; urs.keller@onwd.com (U.K.); joachim.vonzitzewitz@onwd.com (J.v.Z.); 5 Center for Neuroprosthetics and Brain Mind Institute, School of Life Sciences, Swiss Federal Institute of Technology (EPFL), 1015 Lausanne, Switzerland; gregoire.courtine@epfl.ch; 6 Department of Neurosurgery, Lausanne University Hospital (CHUV) and University of Lausanne (UNIL), 1011 Lausanne, Switzerland; 7 Department of Clinical Neuroscience, Lausanne University Hospital (CHUV) and University of Lausanne (UNIL), 1011 Lausanne, Switzerland; 8 Defitech Center for Interventional Neurotherapies (.NeuroRestore), CHUV/UNIL/EPFL, 1011 Lausanne, Switzerland; 9 Department of Rehabilitation Medicine, Erasmus MC, 3000 CA Rotterdam, The Netherlands

**Keywords:** wearable sensor, real-time gait detection, gait analysis, insole pressure sensors, inertial measurement unit, pathological gait, gait rehabilitation, assistive device

## Abstract

Gait analysis has traditionally been carried out in a laboratory environment using expensive equipment, but, recently, reliable, affordable, and wearable sensors have enabled integration into clinical applications as well as use during activities of daily living. Real-time gait analysis is key to the development of gait rehabilitation techniques and assistive devices such as neuroprostheses. This article presents a systematic review of wearable sensors and techniques used in real-time gait analysis, and their application to pathological gait. From four major scientific databases, we identified 1262 articles of which 113 were analyzed in full-text. We found that heel strike and toe off are the most sought-after gait events. Inertial measurement units (IMU) are the most widely used wearable sensors and the shank and foot are the preferred placements. Insole pressure sensors are the most common sensors for ground-truth validation for IMU-based gait detection. Rule-based techniques relying on threshold or peak detection are the most widely used gait detection method. The heterogeneity of evaluation criteria prevented quantitative performance comparison of all methods. Although most studies predicted that the proposed methods would work on pathological gait, less than one third were validated on such data. Clinical applications of gait detection algorithms were considered, and we recommend a combination of IMU and rule-based methods as an optimal solution.

## 1. Introduction

### 1.1. Motivation

Traditionally, performing gait analysis required a dedicated laboratory and expensive equipment, which has limited its scope of applications. Recent advancements in technology have led to reliable, affordable, and wearable sensors for gait analysis that enable its use outside of a laboratory environment and during activities of daily living. One of its primary uses has been in diagnosing walking impairment in people with gait disabilities [1,2,3,4] and inspires control mechanisms of exoskeletons [5,6] and prostheses [7], among other applications [8,9,10,11,12]. More specifically, real-time gait analysis has proven essential in applications necessitating real-time control such as exoskeletons and prostheses, as well as gait rehabilitation involving Functional Electrical Stimulation (FES) [13,14] or Epidural Electrical Stimulation (EES) [15].

Physiological human gait is a quasi-periodic, synergistic process involving the timely actuation of several lower-limb muscles, well-coordinated by neurons in the brain and the spinal cord [5]. Gait disorders and disabilities can arise due to various reasons including amputation of lower limbs, neurological diseases such as Parkinson’s, cerebral palsy and Huntington’s, as well as through stroke or paralysis following an injury to the brain or the spinal cord. This review is particularly motivated by the use of real-time gait analysis in an on-going clinical study (STIMO, ClinicalTrials.gov, NCT02936453): a First-in-Man study to confirm the safety and feasibility of a closed-loop EES in combination with overground robot assisted rehabilitation training for patients with chronic incomplete spinal cord injury (SCI) [16].

We first carried out a systematic review and meta analysis across four major scientific databases (Scopus, Web of Science, Cochrane and PubMed) to identify the current state of the art in wearable sensor-based real-time gait analysis. We then extracted studies that focused on pathological gait and analyzed the most common sensors and techniques used in clinical applications.

### 1.2. Previous Reviews

Before moving into the details of our study, we will briefly discuss our analysis of existing review articles from literature, the results of which are summarized in Table 1. It can be noted from the table that reviews done so far are either specific to a particular category of gait detection method, are not systematic, lack coverage across major citation databases or focus only on wearable sensing. Furthermore, throughout the literature, we noticed the synonymous usage of the terminologies gait detection, gait event detection, and gait phase detection. Although we appreciate the specific difference in terminologies, for the sake of brevity, we will use gait detection to imply gait event and/or gait phase detection.

Among the 11 review studies analyzed, only four of them were systematic reviews. Taborri et al. [17] performed a systematic review on wearable and non-wearable sensors used in gait detection. The study identified various wearable sensors such as inertial measurement units (IMU), insole pressure sensors (IPS), electromyography (EMG) and electroneurogram (ENG), and non-wearable sensors such as opto-electronic systems, force plates, and ultrasonic sensors. The study, however, was limited to sensors and did not provide any review of gait detection methods. Panebianco et al. [18] performed a systematic review covering PubMed, Scopus, and Web of Science. Although the search keywords were not restrictive to rule-based methods, all 17 of the studies involved were limited to rule-based methods. Caldasa et al. [19] performed a systematic review across major databases such as Web of Science, ScienceDirect, IEEE, PubMed/MEDLINE, SCOPUS, CINAHL, and Cochrane, thereby ensuring an exhaustive coverage. However, the review was limited to only artificial intelligence-based gait detection methods using inertial measurements, resulting in only 22 studies that met the acceptance criteria. Chen et al. [20] performed a systematic review focusing particularly on quantifiable gait measures and tangible evaluation techniques that are based on wearable sensors, particularly inertial measurement units (IMU). The study also includes a review of nonlinear analysis techniques such as phase portrait, Poincaré map, Lyapunov exponent (for gait stability assessment), and elliptical Fourier analysis (for gait complexity assessment). The study, however, did not report any real-time gait analysis methods. It can be noticed from these systematic reviews that they are either limited to review of sensors or to a specific category of gait detection method or did not consider real-time gait detection methods. Finally, none of these studies presented information about pathological aspects of wearable sensor-based gait detection.

To the best of the authors’ knowledge, there does not exist a systematic literature review that identifies various wearable sensing options, real-time gait analysis methods, and presents pathological aspects of wearable sensor-based gait detection. We therefore performed a systematic review and meta-analysis across the four major scientific databases mentioned earlier, following the PRISMA (Preferred Reporting Items for Systematic Reviews and Meta-Analyses) standard [21], covering 1262 articles, as will be discussed in the next sections.

**Table 1:** Previous reviews: gaps identified from existing review articles from literature, covering wearable sensor-based gait detection.

References	Focus of Review	Database Covered	Gaps Identified in Existing Reviews	Number of Articles Included
Song et al. [8]	Health sensing techniques with a particular focus on smartphone sensing	Not specified	Not a systematic review, no review of gait detection methods	-
Shull et al. [22]	Clinical impact of wearable sensing	MEDLINE, Science Citation Index Expanded, CINAHL, Cochrane	Not a systematic review, no review of gait detection methods	76
López-Nava and Muñoz-Meléndez [23]	Review on inertial sensors and sensor fusion methods for human motion analysis,	ACM Digital Library, IEEE Xplore, PubMed, ScienceDirect, Scopus, Taylor Francis Online, Web of Science, Wiley Online Library	Not a systematic review, no review of gait detection methods, review limited to inertial sensors	37
Novak and Riener [24]	Sensor fusion methods in wearable robotics	Not specified	Not a systematic review, no review of gait detection methods	-
Vu et al. [25]	Gait event detection methods applicable specifically for prosthetic devices	Scopus, ScienceDirect, Google Scholar	Not a systematic review, review restricted to one category of rehabilitation devices	87
Rueterbories et al. [26]	Review of sensor configurations and placements, and a brief review of gait detection methods	Not specified	Not a systematic review, gait detection methods were reviewed very briefly	-
Perez-Ibarra et al. [27]	Brief review comparing gait event detection methods, sensors used, placement of sensors and subjects involved	Not specified	Brief review, as a subset of the article	18
Taborri et al. [17]	Wearable and non-wearable sensors used in gait detection	Scopus, Google Scholar, PubMed	No review of gait detection methods	72
Caldasa et al. [19]	Artificial intelligence-based gait event detection methods using inertial measurements	Web of Science, ScienceDirect, IEEE, PubMed/MEDLINE, Scopus, CINAHL, Cochrane	Review was limited to only one type of sensor and one type of gait detection algorithm	22
Panebianco et al. [18]	Rule-based methods	PubMed, Scopus and Web of Science	Review was limited to only one category of gait detection algorithm	17
Chen et al. [20]	Quantifiable gait measures and tangible evaluation techniques that are based on wearable sensors	PubMed, IEEE Xplore, ACM Digital Library, EBSCO and Cochrane Library	No review of real-time gait analysis methods	35

### 1.3. Structure of the Report

The remainder of the article is organized into three sections. In Section 2, we describe the methods followed in setting up the systematic review. In Section 3, we present and discuss the results regarding: the search results in general in Section 3.1, gait events and gait phases in Section 3.2, wearable sensors in Section 3.3, algorithms used for wearable sensor-based real-time gait analysis in Section 3.4, and interpretations towards clinical applications in Section 3.5. Finally, in Section 4, we present the conclusions.

## 2. Method: Setting up the Review

### 2.1. Choice of Databases

Haddaway et al. [28] classified scientific literature databases into two categories: Academic Citation Database (ACDB) and Academic Citation Search Engine (ACSE). ACDBs include the traditional Boolean string-based search engines such as Scopus, Web of Science and PubMed, while ACSEs include Google scholar and semantic/natural language-based search engines such as Microsoft Academic Search and Semantic Scholar. We first explored both categories before making a choice.

We considered nine of the most popular ACDBs in our selection process: CINAHL, EBSCO, ACM digital library, IEEE Xplore, Science Direct, Scopus, Web of Science, Cochrane, and PubMed. CINAHL, EBSCO, and ACM digital libraries were not included in the study because of a lack of access to them. We could not include IEEE Xplore since it limits the number of search terms to 15, which is noticeably lower than the number of keywords used in this study (see Table 2). However, this should not impact the comprehensiveness of our study, since IEEE articles are already indexed in Scopus and Web of Science. Science Direct was not included, as a recent update in their search keyword input framework limited us from inputting all our keywords, thereby making it unsuitable for our systematic review. However, this should not impact the comprehensiveness of our study since Science Direct and Scopus share the same database [29] and come from the same parent company (Elsevier). Finally, we decided not to include ACSEs in the review primarily because of deficient repeatability and reproducibility of search results, among other factors [28,30,31,32]. The remaining databases were thus Scopus, Web of Science, Cochrane and PubMed. ACDBs such as Scopus and Web of Science use a selective procedure to safeguard against low-quality or low-impact material being indexed [33], while Cochrane and PubMed are expected to add more clinically relevant studies.

### 2.2. Choice of Keywords for Search

**Figure 1:**
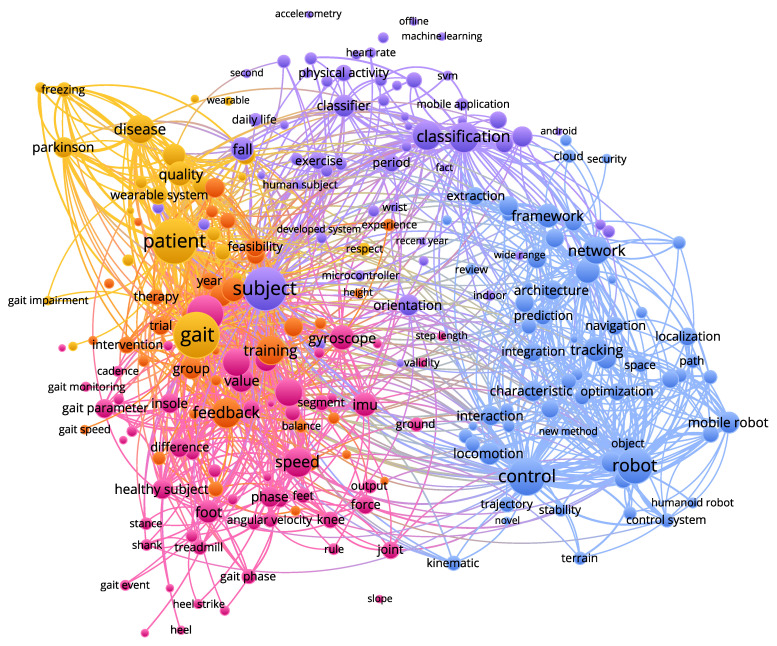
Word cloud showing most frequently occurring keywords present in search results for a search query in Scopus (for instance, see Table 2), visualized by the software VOS-Viewer [35]. The size of each node indicates the relative relevance (based on the frequency of occurrence of keywords) of that topic among the list of articles returned by the search query. Connections between nodes were not used in refining the search terms.

To establish an appropriate search phrase, a pre-search was carried out first, collecting a list of keywords used by gait analysis researchers. In an attempt to find an optimum keyword-combination from the list, we analyzed these keyword-combinations by taking the conducted search results (from Scopus) to VOS-viewer [34], a metadata analysis software. VOS-viewer performs clustering of search results based on title, abstract and keywords of corresponding articles and illustrates the results graphically as shown in Figure 1. This gives us a bigger picture of the nature of articles returned by the search engine for the corresponding choice of keywords. The size of each node indicates the relative relevance (based on the frequency of occurrence of keywords) of that topic among the list of articles returned by the search query. This procedure was iterated and refined several times before arriving at the final search phrase listed in Table 2. We believe that it made the decision-making less subjective and biased. Although this study is not limited to any particular wearable sensor, we included the keywords ‘IMU’ and ‘insole’ (see Table 2) explicitly so as not to miss out articles related to these two types of sensors, while also retaining the word ‘sensor’ in the search phrase to make it inclusive for every other type of wearable sensors.

**Table 2:** Keyword combination used for search in Scopus database which resulted in 697 articles (see Figure 2). The same keyword combination was used in the other databases as well, except adapting syntax to individual search engines.

realtime OR “real time” OR online
AND
gait OR walking OR locomotion OR “lower limb” OR “lower body” OR
leg OR “lower extremity”
AND
analysis OR detection OR evaluation OR assessment OR estimation OR
reconstruction OR tracking
AND
wearable OR portable OR mobile
AND
sensor OR “inertial measurement unit” OR accelerometer OR IMU OR gyroscope OR
insole OR in-sole

### 2.3. Carrying Out the Review in a Systematic Manner

**Figure 2:**
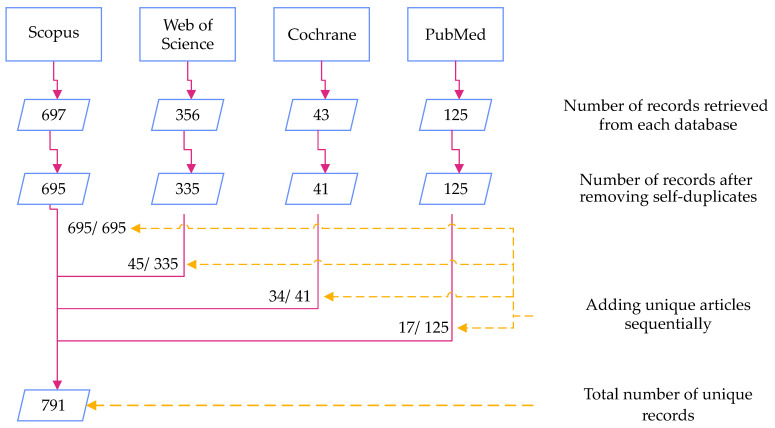
Collecting unique articles from the databases was carried out sequentially, starting with Scopus where 697 articles were extracted and of which 695 unique records were identified. Out of the 335 unique records identified from Web of Science, 290 articles already appeared in the results from Scopus and hence the remaining 45 unique records were added. Similarly, 34 from Cochrane and 17 from PubMed were added to the list of unique records.

**Figure 3:**
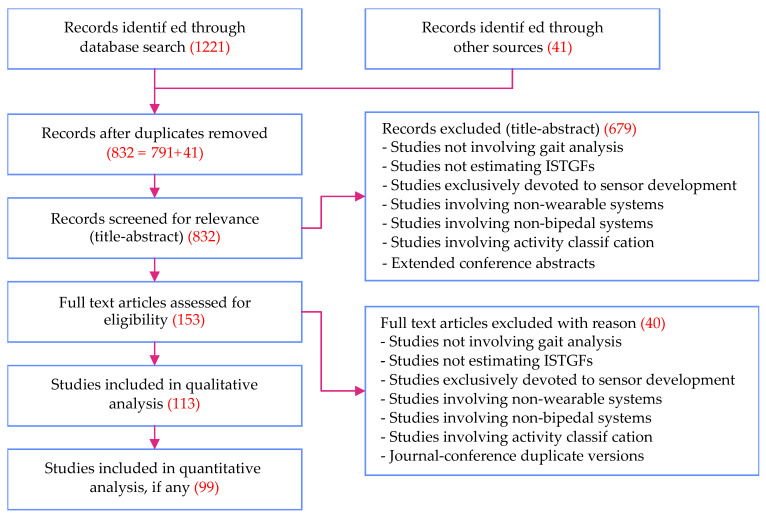
PRISMA flow diagram illustrating the screening procedure. Reasons for exclusion and the number of articles retrieved at each stage are indicated in red. In addition, 832 articles were left after removing duplicates. Out of those, 679 articles were eliminated through title-abstract screening, based on a set of exclusion criteria as listed in the PRISMA flow diagram. The remaining 153 articles qualified for full-text screening, of which 40 were excluded and the remaining 113 qualified for full-text review. Out of these, 99 articles were also used for quantitative analysis. ISTGF—intra-stride temporal gait feature.

In this study, we followed the PRISMA standard for systematic review. A total of 1262 articles were part of the review, including 1221 articles retrieved from the four databases (see Figure 2) and an additional 41 articles added later from bibliographies of the former. Figure 3 shows the PRISMA flow diagram illustrating the screening procedure followed. The screening procedure was performed independently by the two lead authors of our study. Studies for which the authors had difference of opinion on their exclusion (such as studies that were at the border line of the exclusion criteria) were mutually discussed and decided upon. Although assessment of paper quality is not mandated by the PRISMA standard, the authors’ choice of databases ensured that low-quality and low-impact material was not considered.

We classify the most commonly used gait features into intra-stride (within stride) and inter-stride (between strides) features, and into temporal and spatial features. Intra-stride features are of higher granularity, looking at the gait in more detail, while inter-stride features are of lower granularity. The choice of each depends on the application. Examples of each category are listed in Table 3.

In this review, we focus on intra-stride temporal gait features (ISTGFs), the rationale being that inter-stride features can be obtained from related intra-stride features (e.g., stride duration and cadence can be obtained from temporal information of consecutive heel strikes). We therefore believe that detecting sufficient ISTGFs is sufficient for insightful gait analysis in practice. In addition, ISTGFs are necessary for real-time control applications such as in the clinical study [16] that has served as a particular motivation for this review. Hence, studies not estimating the ISTGFs (such as indoor localization algorithms, gait reconstruction methods and activity classification methods) were excluded from this review.

Other major reasons for exclusion are listed in the PRISMA flow diagram (see Figure 3). Studies not involving bipedal systems, studies not involving wearable systems, and studies devoted purely to (wearable) sensor development are directly excluded. When both a conference version of an article and its extended journal version appeared in our search results, the conference version was excluded. In a rare observation, we noted two sets of nearly duplicate conference publications from the same set of authors [7,36,37,38]. In this case, only the latest ones were considered for further review. Finally, if an article was found to compare, list, or review multiple gait event detection methods introduced in other studies, the original studies were included in the review rather than the former.

## 3. Results and Discussion

### 3.1. Search Results

The systematic review resulted in the identification of 1262 studies, as indicated in Figure 3. After removal of duplicates, we were left with 832 unique studies. Out of these, 113 and 99 qualified for qualitative and quantitative analysis respectively. Studies that underwent qualitative analysis influenced our discussions while quantitative analysis resulted in the extraction of metadata that was presented throughout the paper. The 14 studies not included in the quantitative analysis did not contain all the necessary metadata and therefore did not contribute directly to the metrics presented.

Review studies cited in Section 1.2 have an average of 30 full-text articles per review, with a maximum of 76 by Shull et al. [22]. The present work is thus one of the most comprehensive reviews on the subject.

### 3.2. Gait Events and Gait Phases

Irrespective of the type of wearable sensors used and the type of real-time gait analysis methods followed, here we will briefly discuss the two major ISTGFs from literature: gait events and gait phases.

Researchers followed different terminologies for various gait events. Some authors prefer to use initial contact (IC) (or sometimes touch down) instead of being more specific as to whether the contact is with heel strike (HS) or with toe strike (TS). Although HS is most often the obvious initial contact in unimpaired gait, it is not necessarily the case with impaired gait. For instance, initial contact in the case of toe walking could be TS instead of HS. Similarly, some authors prefer end contact or foot off instead of using the more explicit terminologies: toe off (TO) or heel off (HO). On the other hand, for gait phases, researchers tend to use consistent terminology to decompose stance and swing: loading response, mid-stance, terminal stance, pre-swing, initial swing, mid-swing, and terminal swing [6,39,40,41,42].

**Figure 4:**
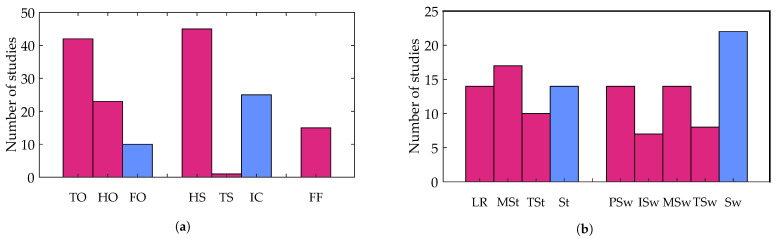
(**a**) Distribution of studies based on the detected gait events; (**b**) distribution of studies based on the gait phases identified. Gait events/phases reported with greater (temporal) specificity are shown in magenta while gait events/phases reported with less specificity are shown in blue. For instance, initial contact (IC, blue) is not specific as to whether the contact is with the heel or toe while heel strike (HS, magenta) and toe strike (TS, magenta). TO—toe off, HO—heel off, FO—foot off, HS—heel strike, TS—toe strike, IC—initial contact, FF—foot flat, LR—loading response, MSt—mid-stance, TSt—terminal stance, St—stance, PSw—pre-swing, ISw—initial swing, MSw—mid-swing, TSw—terminal swing and Sw—swing.

A detailed count of gait events and gait phases used in the resulting studies is shown in Figure 4a,b respectively. TO and HS are the most widely identified gait events irrespective of the type of sensor used. A total of 42 studies detected TO while 45 detected HS, suggesting the high relevance and ease with which these events can be identified from gait signals. Among the gait phases, swing (22 studies) was the most widely identified gait phase followed by mid-stance (17 studies).

### 3.3. Sensors

**Figure 5:**
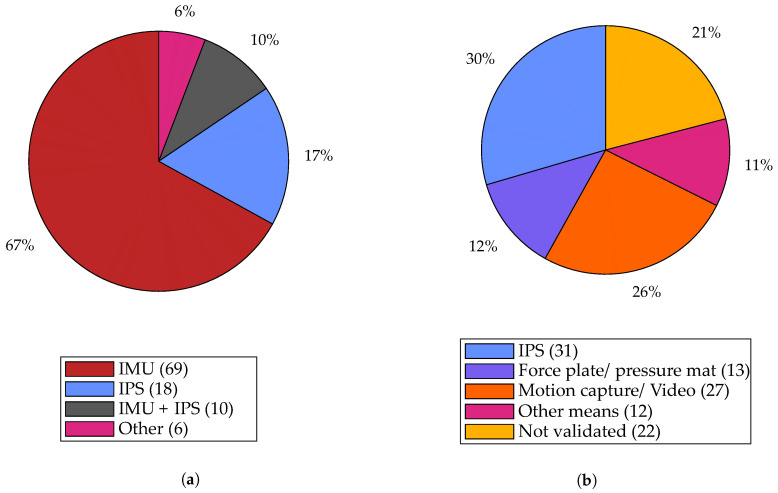
(**a**) Distribution of studies based on the type of wearable sensors used; (**b**) distribution of studies based on the type of sensors used for ground-truth validation of IMU-based gait analysis. Absolute number of studies in each category is listed within parentheses. IMU—inertial measurement unit, IPS—insole pressure sensor, EMG—electromyography sensor.

In order to have an overview of relevant wearable sensors available on the market, a survey of off-the-shelf devices was conducted. Wearable sensors identified include primarily IMUs, insole pressure sensors (IPS), electromyography (EMG) sensors, goniometers, inclinometers, electromagnetic trackers, and stretch sensors. However, only three main types of wearable sensors could be identified among the 99 studies that featured in the quantitative analysis of our review: IMU, IPS, and a combination of the two. The distribution of sensors used is shown in Figure 5a and discussed in the following sections. A check was performed on the Scopus database to ensure that our explicit addition of search terms regarding IMU and IPS were not heavily biasing the results. In fact, only 98 additional studies were found compared to the 725 identified without the search terms, and we therefore conclude that their explicit addition was not responsible for the dominance of these sensor types.

#### 3.3.1. Inertial Measurement Units

Inertial measurement units (IMUs) are sensors combining accelerometers and gyroscopes to measure linear acceleration and angular velocity of the body to which it is attached. Optionally, it also comes with a magnetometer that can estimate the magnetic north based on the earth’s magnetic field and is sometimes called an inertial-magnetic measurement unit. As shown in Figure 5a, we notice that IMUs are the most widely used sensors with 77% of studies using it either alone (67%), or in combination with IPSs (10%).

Appropriate sensor placement often simplifies or even eliminates any calibration required for gait detection algorithms. Gyroscopes are invariant to translation in position [39] since the angular velocity of a rigid body is the same at any point along the body (assuming the orientation of the sensor remains the same with respect to the body segment). They are also unaffected by gravity and are less prone to noise. Accelerometers, on the other hand, are reported to be more noisy, subject to the influence of gravity and sensitive to both position and orientation. The sensitivity to sensor orientation is typically avoided by considering the norm of acceleration instead of acceleration along individual axes. The influence of gravity is often used to estimate the orientation of the sensor with respect to the earth frame of reference (in combination with additional constraints such as the Earth’s magnetic field).

**Figure 6:**
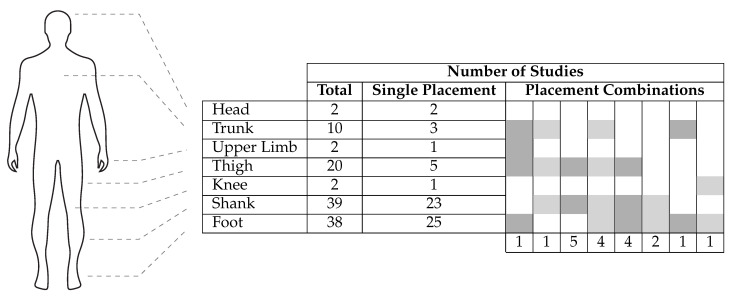
Number of studies using inertial measurement units (IMUs) that placed the sensor(s) on specific anatomical locations. Single placement contains studies where sensor(s) were placed only in one anatomical location. Placement combinations’ columns indicate studies where sensor(s) were placed in more than one location. Each relevant location is marked by a shaded cell and the number of studies using this combination is indicated at the bottom of the column. The total indicates the sum of studies where the sensor(s) were placed on that given anatomical location.

With IMUs, various possibilities for sensor placement exist and researchers have tried a number of approaches for gait-related studies, placing IMUs on different body segments or combinations thereof. The approaches are quantified in Figure 6. Among the studies which used IMUs, the shank was the most widely preferred lower-body segment for gait analysis (with 39 studies) closely followed by the foot (with 38 studies).

Although these numbers give us a better understanding of the preference followed in literature, they alone do not necessarily tell us whether these segments are the ones that provide the richest information of gait or if preferring these segments over others makes it easy to identify ISTGFs from gait data. Some researchers attempted to give a clearer answer to these questions. Li et al. [43] compares IMU signals from the thigh, the shank and the foot based on what they call, the “energy of acceleration,” which is the norm of raw acceleration minus gravity. They argue that the “energy of acceleration” (when inspected graphically) appears to be relatively more “stable” (i.e., constant) in the foot compared to the other two body segments and hence recommend IMU placement at the foot. Mazilu et al. [12], in the context of freezing of gait, reports 98% or more detection performance for all three body segments, suggesting that the question of optimal sensor placement is irrelevant in the context of freezing of gait. Jasiewicz et al. [44] report that, upon comparison of three rule-based gait event detection methods, the method based on foot angular velocity and linear acceleration was significantly more accurate than that of the method based on the shank for spinal cord injured (SCI) subjects. Taborri et al. [45] made a similar observation for a hidden Markov model (HMM)-based classifier. They report that the accuracy of a HMM-based classifier for gait event detection was better when the angular velocity of the foot was used rather than of the thigh or the shank. The relatively high preference for the foot could also be justified by the results from neuro-behavioral experiments suggesting that limb endpoints are the primary variables used to coordinate locomotion in animals and humans [15]. Bejarano et al. [46] analyzed four signals—linear acceleration in forward and vertical direction, angular velocity, and segment angle normal to the sagittal plane—for the thigh, the shank, and the foot. Acceleration components were discarded (after a preliminary investigation) due to noise and vibrations while the root mean square error between each cycle (as well as the average) was computed for the other two signals. For both angular velocity as well as segment angle, sensors placed on the shank were identified with noticeably low root mean square error and hence the authors recommended using the shank as the preferred location for IMU placement.

Much like the inter-segment IMU placement problem just discussed, a user could also place the IMU at a different position and orientation within a given body segment, each time it is attached to the body. Such intra-segment differences may result in undesirable variations across data sets and across subjects. This is typically avoided by using a mount/socket so that the sensor falls into the same location every time it is inserted. Anwary et al. [47] suggests that the optimal location for IMU placement on the foot is the medial arch followed by the Achilles tendon.

Raw signals from IMUs are noisy, particularly the accelerometer signals, and thus filters are widely used. Meta-analysis on preprocessing filters used in the case of IMUs revealed that 39 out of 69 studies used at least one preprocessing filter, 31 of which used a low-pass filter among which 15 used the Butterworth low-pass filter. Note that preprocessing filters add to the latency in data processing, which is undesirable in a real-time system.

The orientation measured by an IMU is often useful in gait analysis. One way to estimate the orientation is by integrating the angular velocity from the gyroscope. However, due to gyroscopic bias, such an approach is prone to drift from numerical integration. Under static conditions, accelerometers can be used to estimate the inclination with respect to the gravity vector, while magnetometers can be used to estimate orientation with respect to the Earth’s magnetic field (magnetic North). Since acceleration measurements from accelerometers are prone to noise, estimating the orientation outside of static conditions is not accurate from instantaneous sensor data. Magnetometers, on the other hand, are sensitive to external magnetic fields. Sensor fusion methods combine the information from accelerometers, gyroscopes, and magnetometers (or a subset of these) to provide a better estimate of the orientation of the sensor. Kalman filter-based and complementary filter-based methods are the two most popular sensor fusion methods used for estimating orientation from IMUs. Casamassima et al. [48] compared these two methods based on accuracy, computational cost, and energy efficiency and concluded that the Kalman filter-based method was their preferred choice. Two of the most popular methods using the complementary filter are proposed by Mahony et al. [49] and Madgwick et al. [50]. Cirillo et al. [51] observed that a version of the extended Kalman filter-based method that offers similar performance to the two took approximately one order of magnitude more time to process a sample in the MATLAB/Simulink environment and two orders of magnitude more time in an embedded system environment. Overall, Kalman filter-based methods are known to be more accurate but computationally demanding, while complementary filter-based methods are known to be computationally light and fairly accurate [52].

Within the context of walking, accuracy of orientation (and position) estimation using IMUs can be enhanced using additional constraints from the foot–ground interaction. The zero velocity update (ZUPT) algorithm or one of its variants are typically used to compensate drift. The algorithm exploits the fact that, during a part of the stance phase, the stance foot is quasi-static. During this moment, the linear and angular velocity of the foot is assumed to be zero and the drift errors due to integration are reset. Yang et al. [53] estimated the stance duration from thresholds set on both angular velocity and acceleration, which helped in correctly applying the ZUPT. Skog et al. [54] compared four different detectors to identify the zero velocity interval—”acceleration moving variance detector, the acceleration magnitude detector, the angular rate energy (ARE) detector, and a generalized likelihood ratio test detector, referred to as the SHOE”—and concluded that both ARE and SHOE performed with very high accuracy. Inspired by this, Refs. [48,55] used a threshold on the ARE to estimate the ZUPT interval by hypothesizing that the IMU is stationary when ARE is below the threshold. Other variants of ZUPT are also attempted [56,57]. In [57], the foot inclination angle, obtained by integrating the gyroscope signal, was reset to zero during the stance phase based on input from an IPS.

Often, sensors regarded as the gold standard, providing ground-truth information, are used to validate results obtained from IMU-based gait analysis. Figure 5b illustrates the distribution of those sensors used in validating IMU-based gait analysis. Among the studies which used IMU-based gait analysis, it can be observed that IPSs are the most widely used sensors for validating the results with 31 studies compared to 27 studies using motion capture/video. In addition, they are the only wearable sensor to be used for validation.

#### 3.3.2. Insole Pressure Sensors

An insole pressure sensor (IPS) measures the pressure distribution at the foot, which is widely used to estimate the COP along with other gait parameters such as step count, duration of the gait cycle, swing duration, stance duration [58], and foot–ground interaction events (such as HS or TO). IPSs are available in different variants based on optoelectronic sensors, force-sensing resistors (FSRs), capacitive sensors, and piezoelectric sensors, which are based on polyvinylidene difluoride (PVDF) films [59]. PVDF films lack durability, although they are reliable and inexpensive. FSRs, on the other hand, are highly durable, flexible, and inexpensive. The reliability of FSRs is low when estimating the magnitude of force in real-time, but FSRs perform well in detecting the temporal information such as the instant of application of force, as shown by [59], and hence are good candidates for real-time gait event detection. Delgado-Gonzalo et al. [60] report that IPSs have a short lifespan, although the claim is not adequately validated. We noticed in our review that 38 out of 59 studies are from 2014 or later, possibly suggesting that IPSs have become more reliable over the years.

IPSs are the second most widely used wearable sensor with 57% of the studies using it either for sensing (28 studies, see Figure 5a) or validation (31 studies, see Figure 5b). It is the only wearable sensor to be used in validation studies, which arguably makes it the gold standard in wearable sensing [61]. IPSs are often considered as an alternative to force plates in validation studies due to several advantages including cost factor, wearability, and unconstrained movement that allows natural gait in both indoor and outdoor environments. Despite these advantages, there are some constraints to consider. IPSs are typically placed inside the shoe and are thus subject to pressure between it and the foot, which can lead to non-zero pressure readings even when the foot is in swing phase [42,59]. Although IPSs are comparable to force plates when it comes to estimation of temporal features, using them for real-time ground reaction force estimation is not recommended since it takes a considerably longer time to reach the set value compared to a force plate [59].

Unlike IMUs, sensor placement is not a challenging problem for IPS. While the user could place the IMU anywhere within the body segment of interest, IPSs are almost always placed in the subject’s shoe, making them fall into the same position with respect to the foot. The traditional approach to place FSRs within an IPS has been to place them at specific hotspots such as the heel, toe, first, and fifth metatarsals. Such IPSs require the correct foot size of the subject so that FSRs are aligned with the correct hotspots. Senanayake et al. [42] reported errors in measurement owing to subjects involving varying foot sizes (6–11) while the IPS was at a fixed size (eight). Lin et al. [62] reported robustness against this offset, caused by the size mismatch of the IPS with the foot, by using the derivative of pressure signals. The authors used an array of 48 pressure sensors, giving a better resolution than the conventional approaches, which place a few FSRs at carefully chosen hotspots. The authors of [63,64] followed a similar approach using a pressure signal from the IPS and its first derivative while using an IPS with 64 optoelectronic sensors. With IPSs getting better in resolution, the approach is shifting towards packing as many sensors within the insole as possible so as to collect data throughout the feet and identify the hotspots not at the hardware end, but later at the software end during signal processing. When used in real-time, this demands more bandwidth for communication and computational power to process the additional information.

In contrast to IMUs where 39 out of 69 studies at least used a preprocessing filter, only six out of 28 studies related to IPS used any sort of preprocessing/filtering. Instead, these studies relied directly on the raw signal from the IPS, likely contributing to shorter latency, an advantage when it comes to real-time systems.

#### 3.3.3. Combination of IPS and IMU

A new kind of product that is emerging in the wearable sensor market is an IPS combined with an IMU, such as Moticon Science from Moticon GmbH, Munich, Germany, Stridalyzer from Retisense, Bangalore, India and Arion Wearable from ATO-GEAR, Eindhoven, The Netherlands. Such a set up allows combining the advantages of both types of sensors. Ten out of 52 studies used a combination of IPS and IMU (in addition to the studies that used IPS separately for validating IMU-based gait detection results). Depending on the product, it is possible that the position of the IMU is fixed relative to the IPS, thereby minimizing the errors caused by differences in IMU placements between and within segments as well as across data sets and subjects.

#### 3.3.4. Other Wearable Sensors

Other wearable sensors used for gait detection include Electromyography (EMG) sensors, rotary encoders, laser range finders, flex sensors, and capacitive shank orthosis. Other than EMG sensors, all the other sensors measure kinematics. EMG sensors, on the other hand, measure electrical activity in muscles, which gives them an inherent advantage that signals appear earlier than the corresponding movement from muscle activation [5]. Fleischer et al. [65] reports that EMG signals appear 20–80 ms before the resulting contraction begins. This should contribute to early sensing and hence decrease latency in control. Farmer et al. [66] presented an auto-correlation model that takes EMG signals as input to predict the ankle angle, which is claimed to predict around 100 ms in advance. Despite this advantage, which is particularly important in real-time gait detection, it is interesting to note that only one out of the 99 studies used EMG sensors. This could partly be because of usability constraints: the skin is typically prepared by shaving body hairs and applying abrasive gel to increase signal-to-noise ratio, and the sensor is taped to the skin in order to keep the contact constant and reduce motion artifacts. It could also be owed to the fact that EMG signals require more preprocessing/filtering and EMG signals of persons with certain impairments (primarily neurological deficits) can be weak and hard to interpret. Furthermore, EMG-related parameters are subject-dependent and can change regularly due to varying conditions of the skin and body state, such as sweat. Correct sensor placement is also non-trivial and requires some training because the sensor should be placed as close as possible to the belly of the appropriate muscle. This approach may be less appropriate for lay users thus limiting its application. Evaluation of EMG patterns are mostly done using classification algorithms and less often using physiological models [65].

### 3.4. Real-Time Gait Analysis

We classify various gait analysis methods identified from literature into three main categories: time domain-based, frequency domain-based, and time-frequency domain-based. Table 4 shows various real-time gait analysis methods used by researchers based on this classification, some of which are discussed in greater detail in the subsequent sections.

In summary, we observe that the rule-based methods are the most popular, with a majority of the studies using it, likely due to their simplicity and intuitiveness compared to other computationally expensive solutions. Phase portraits and adaptive oscillators are among the limited number of methods noted for continuous gait phase estimation. Wavelet transforms are seen as more suitable for fast motion transitions, and the method may serve as a better candidate in gait phase estimation than adaptive oscillators.

Performance of the different methods is not compared quantitatively since evaluation criteria varied from one study to another, which makes an objective comparison difficult. For instance, a study that was intended for impaired gait but was tested only with unimpaired subject can present better results that need not translate to impaired subjects.

#### 3.4.1. Rule-Based Methods

Rule-based methods are the most widely used gait detection technique, employed by 63 out of the 99 studies. The wider adoption of the method could be attributed to their simplicity, intuitiveness, and less computational complexity involved (and hence less latency in the real-time processing).

One way of implementing a rule-based method is by setting a threshold on the raw or processed signal from the IMU (for instance, on the acceleration, angular velocity, segment orientation angle, or joint angle). Rule-based methods often employ multiple rules built on conditional statements (typically, if-else logic) that are connected using inequality constraints or logical AND/OR operators.

Sometimes, threshold-based techniques are replaced with peak detection techniques. One disadvantage of peak detection is that the presence of a peak can be confirmed only after both the rising edge and the falling edge appear. This may introduce a delay in gait event/phase detection depending on which part of the peak the event/phase temporally overlaps with. Maqbool et al. [7] followed such an approach wherein the shank angular velocity in the sagittal plane is used with a window of 80 ms before confirming the peak, while Ref. [37] additionally used accelerometer signals.

Instead of using predetermined thresholds, Ref. [58] proposed an adaptive threshold-based method which automatically computes and updates the threshold in real-time. This is done through what is called the “dynamics of sensor data”, defined as a function of linear acceleration and angular rate, averaged over the last five data samples. The adaptive threshold is used to distinguish between swing and stance phase.

Rule-based approaches are also popular in the case of IPSs, with several studies using threshold or peak detection based approaches on IPSs, either to distinguish between stance phase and swing phase or between multiple gait phases [57,67,68,69,70]. Lin et al. [62] set a threshold on the first derivative of the pressure sensor data in identifying HS and TO and reports that it makes the detection robust against spurious signals, offset variation between IPSs and between-subject variations. Hanlon et al. [71] used a similar approach of setting a threshold on the derivative of the pressure sensor while additionally using a threshold on the accelerometer data along with its first and second derivative.

Rule-based methods are often implemented as finite-state machines (FSM). Pappas et al. [57] reported an FSM that considers four states: swing phase, stance phase, HS, and TO. Seven transitions were defined between these four states based on input from the IPS and the foot pitch angle. The IPS used three FSRs, one each on the heel, and first and fourth metatarsals. The FSRs were used as foot switches to identify if and when weight was applied at these hotspots. It was the only study that considered both stroke and spinal cord injured subjects. The latter were able to walk short distances with or without crutches, but no ASIA impairment scale (AIS) score was mentioned. The study reported above 99% detection reliability for both unimpaired and pathological gait, with detection delay always less than 90 ms. The method is often considered as a benchmark in literature for gait event detection.

Lambrecht et al. [72] used the same to benchmark the performance of three versions of peak detection-based FSM implemented by them. The three methods differed in their input signals, which were chosen from: shank angular velocity, shank segment angle, ankle joint angle, heel linear velocity, toe linear velocity, shank position, foot angular velocity, and foot angle. The methods were otherwise identical in that the state transitions were defined from TO to mid-swing to IC to foot-flat to HO and back to TO. Although the study reported better performance, it is to be noted that the data were extracted using motion capture and is therefore hard to replicate using a wearable sensor (such as linear velocity and position). Hence, the corresponding FSMs may not be easily transferable to a system based on wearable sensing. Furthermore, a direct quantitative comparison between [57] and [72] would be questionable since the data-set used by the former involved both unimpaired and impaired gait while the one used by the latter only involved unimpaired subjects walking on treadmill.

#### 3.4.2. Fuzzy Inference System

An advanced version of the rule-based technique is the fuzzy inference system (FIS). Instead of using thresholds to specify binary states (true or false scenarios) to decide state membership, an FIS fuzzifies the input variable and provides a continuous map between input and output variables based on a systematically designed rule base. González et al. [73] fuzzified the input from pressure sensors placed at the heel, the hallux, and the first and fifth metatarsals into fuzzy variables and defined a rule base whose outputs are gait phases. Senanayake et al. [42] followed a similar approach of fuzzifying four FSR variables while also using the knee angle, obtained from IMUs at the thigh and the shank, as the fifth fuzzy variable. However, the use of additional sensors appears to be counterproductive since a quick comparison of the latencies reported by both approaches reveals that the former (latency less than 77 ms) performed better than the latter (latency less than 300 ms).

One disadvantage of FIS is that it requires the state membership functions to be set by the user [74] and then be adapted each time to a new subject or data set for optimal performance, similarly to what is done with thresholds as discussed in Section 3.4.1. The adaptive neuro-fuzzy inference system (ANFIS) provides a workaround which combines the benefits of artificial neural networks (ANN) and FIS by letting the nonlinear membership functions be learned through the neural network, provided that sufficient training data sets are available. Lauer et al. [74] combined ANFIS with a subtractive clustering method to identify state membership functions followed by a supervisory control system (if-then rules) to prevent gait events from being identified in the reverse order. The subtractive clustering algorithm provided a quick method of estimating the minimal number of clusters required, and these clusters formed the initial shape of the state membership functions. A similar two-level approach using FIS with a supervisory function was also implemented by [13].

#### 3.4.3. Machine Learning

Machine learning (ML) methods are the second most widely used gait analysis technique, with 19 out of 99 studies using them. ML approaches have been gaining popularity in recent years as 18 out of 19 studies are from 2012 or later. The Hidden Markov model (HMM) is the most favored with nine out of 19 studies using this approach. Abaid et al. [3] used angular velocity of the foot in the sagittal plane as input to the HMM, while Ref. [45] used angular velocity of the thigh, the shank and the foot in the sagittal plane. In both cases, FSR based IPSs were used for creating a labelled data set, which is necessary for training the model. Chen et al. [75] used inputs from both IPSs and accelerometers and used a third order fast Fourier transform followed by a principal component analysis for feature generation which was then fed to a support vector machine classifier to identify the gait phases. The study considered five ISTGFs and reported a 97.26% success rate for initial contact. Overall, machine learning techniques reported noticeably high accuracy with 10 out of the 15 studies (which reported at least some quantitative metrics) reporting above 91% accuracy. It is also interesting to note that 11 out of 19 studies detected at least four ISTGFs, which is much higher compared to most of the rule-based methods discussed in Section 3.4.1.

#### 3.4.4. Phase Portrait

Among all the algorithms that were listed in Table 4, only a few studies used continuous gait phase estimation methods. The idea of continuous gait phase estimation is to have a variable keeping track of the progress of gait, continuously and bounded within the gait cycle. Quintero et al. [76] estimated continuous gait phase in real-time from the phase portrait of the hip angle and its derivative. Here, the phase portrait angle of the hip is considered as the continuous gait phase variable—placing the hip angle on the horizontal axis and its derivative on the vertical axis, a phase portrait angle is the angle subtended on the horizontal axis by the line joining the origin to a point on the phase portrait. The hip was chosen based on a more extensive study (although offline) carried out by [77], which reports that the phase angle obtained from the phase portrait of the hip is linearly and monotonically increasing, and bounded, even under perturbations. The phase portrait was scaled by a factor estimated by the ratio of difference in maximum phase angle and minimum phase angle to the difference in the first derivative of the same, so as to improve the monotonicity and linearity. These properties were further improved by filtering, at the expense of some delay. Although the method performed well in unimpaired subjects [76] and the offline analysis reported robustness to perturbations [77], it remains to be seen how the method would work with pathological gait in real-time.

#### 3.4.5. Adaptive Oscillators

An adaptive oscillator (AO) is a frequency domain method that can synchronize to any periodic or pseudo-periodic input signal without any preprocessing [78]. Yan et al. [79] used peak detection to identify a gait event, based on the occurrence of a desired bio-mechanical event (e.g., max hip flexion angle, heel strike). This is used to mark the initialization of a new gait cycle, following which continuous phase estimation of the current gait cycle is carried out using adaptive oscillators. Chen et al. [6] developed a robust adaptive oscillator-based gait phase estimation which is reported to be working robustly even for abnormal gait. HMM was used for gait event detection which in turn was fed to AOs, instead of feeding the entire gait signal continuously. The robustness, according to the authors, is due to the fact that gait events were the only information needed to achieve synchronization which minimized the influence of gait abnormality on the algorithm.

#### 3.4.6. Wavelet Transform

**Figure A1:**
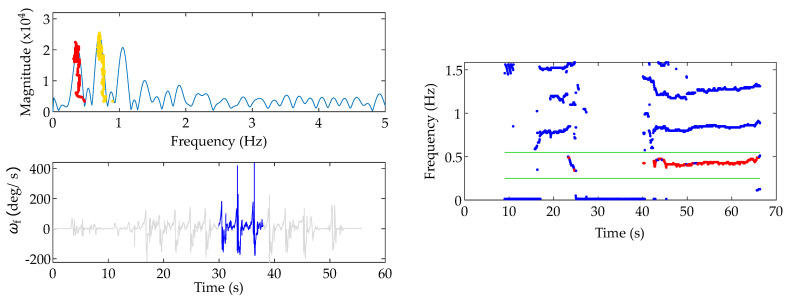
An illustration of the STFT (short-time Fourier transform) implementation in real-time. In the lower left plot, the sagittal plane angular velocity (*ω*_f_) of the foot is shown in grey. The window of sample used during the snapshot (at around time *t* = 38 s) is highlighted in blue. The upper left plot shows the frequency spectrum corresponding to time *t* = 38 s. The red and yellow dots represent the paths traced over the duration by the peaks corresponding to the first harmonic (stride frequency) and the second harmonic (step frequency), respectively. In the right plot, the time-frequency domain output (of the entire data set) from an offline implementation of STFT is shown in blue dots while the corresponding output from the implementation in real-time (for the first harmonic) is shown in the red dots. Note that the sampling interval is slightly irregular in the real-time implementation due to computational complexity associated with STFT. It can be observed that there is a clear separation between the first, second, and third harmonics (the green lines border the first harmonics, which corresponds to the stride frequency).

**Figure A2:**
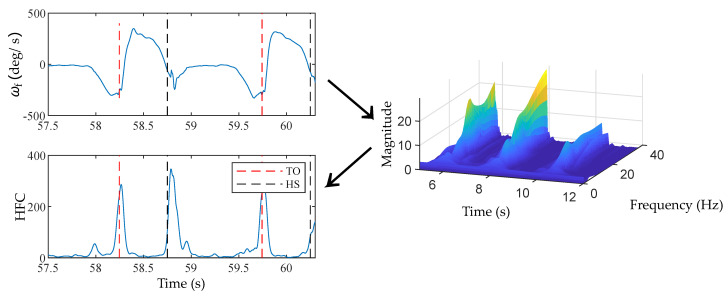
An implementation of wavelet transform for gait event detection. Sagittal plane angular velocity (*ω*_f_) of the foot (top left plot), which is in time domain, was first transformed into time-frequency domain using wavelet transform (right plot). Then, the high-frequency region was condensed into time domain (bottom left plot) by evaluating the cross section area of the magnitude of wavelet transform. A real-time implementation of the method was limited by computational complexity. HFC—high frequency content.

Wavelet transform (WT) is a time-frequency domain method that uses basis functions localized in both the time and frequency domain, through so-called wavelets analogous to sinusoids in a Fourier transform (see Figure A1 and Figure A2 in Appendix A for a detailed description of WT). Features that are identifiable in the frequency domain can therefore be localized in the time domain, for example characteristic high-frequency content during heel strike. Aminian et al. [80] reported that TO and HS events consist of combined features that can be well resolved in the time-frequency domain. They identified distinctive features in the shank angular velocity involving some medium- and high-frequency content with sharp characteristic peaks. A discrete wavelet transform (DWT) with fifth-order Coiflet wavelets was used to enhance gait events in the signal, thereby enabling easier identification of global maxima corresponding to the gait events. This identification was followed by customized rules that found specific peaks in the time domain to confirm TO and HS. Coiflet wavelets were chosen because they resemble characteristic peaks observed in the angular velocity signal. Although the study reported accurate temporal estimation of TO and HS, it should be noted that it was only tested on unimpaired subjects and implemented for offline analysis.

### 3.5. Towards Clinical Applications

#### 3.5.1. Sensor and Algorithm Choice

The motivation of most studies to develop real-time gait analysis techniques was to apply them in gait rehabilitation. Although many of the studies anticipated their proposed methods to work on pathological gait, less than one-third of studies included impaired/pathological gait for validation (31 studies out of 99). When considering clinical applications, the algorithm performance is only one of equally important requirements. The approach must also use sensors that are durable, easy to manipulate, and require a relatively low setup time—in other words, the approach should be simple and user-friendly. Finally, the algorithm must be validated on a sufficient sample of the target population in order to to take into account the unique characteristics of the target impairment and to account for the higher inter-subject variability present in impaired gait.

In Section 3.3 and Section 3.4, we concluded that IMUs are the most common sensor and that rule-based methods are the most common algorithm type, when considered individually. Machine learning approaches are the next most common, under which we combine the hidden Markov model, support vector machine, and Bayesian approaches amongst others. In Table 5, we consider studies that were validated at least on unimpaired subjects (84 out of 99), since we deem these more relevant when it comes to applications, compared to studies that were not validated on subjects at all. The studies are listed according to the method used. We then quantify the number of studies using IMUs for each algorithm type and notice that IMUs remain dominant for both rule-based and machine learning approaches with 75% and 87%, respectively. This does not come as a surprise as they are good candidates for clinical applications: they can easily be placed/removed on/from the relevant body segment and avoid mechanical stress relatively well during typical use. IPSs, the second most popular sensors, are more cumbersome as they must be placed in the shoe and taken out for recharging. For persons with impaired hand-function, this is a point that cannot be neglected. For certain gait impairments where orthopaedic insoles are prescribed, the additional sensorized insole may cause discomfort or provide skewed signals. Lastly, they are subject to repeated mechanical stress, making them considerably less durable.

Improved usability and versatility can be observed with rule-based approaches compared to machine learning. In fact, only 11% of studies using a rule-based method required the placement of more than one IMU per leg, in contrast to 53% of machine learning approaches. This means applications using the rule-based methods typically require a less complex sensor setup, which would be preferable to the end-user. Furthermore, in 84% of studies using rule-based methods, the approach could be used independently for one leg or the other, compared to 47% for machine learning. This means that rule-based methods can be more easily tailored to specific use-cases, such as providing unilateral assistance.

Validation of rule-based and machine learning methods was done on 485 and 138 unimpaired subjects, respectively, which is an average of roughly nine subjects per study for both categories. Impaired and unimpaired gait, however, can vary significantly and thus these numbers speak little towards clinical applications. As stated previously, less than one third of studies were found to validate on impaired subjects, which mirrors findings by Perez et al. [27] that not many real-time gait detection algorithms are validated on populations with gait impairments. Despite this fact, literature and our previous observations are pointing towards IMUs and rule-based methods as primary candidates for clinical applications.

#### 3.5.2. Impaired Gait Considerations

IMUs and rule-based algorithms are the preferred option amongst the studies that validated on impaired subjects. These studies are listed exhaustively in Table 6 and the combination amounts to 67%. We categorized the target impairments into two classes based on how they were presented in the respective studies—first, generally diminished ambulatory function, where gait is impaired due to general degeneration of the locomotor system, such as with Parkinson’s disease, osteoarthritis, Huntington’s disease, diplegic cerebral palsy (CP), ageing, or spinal cord injury (SCI). Second, unilateral loss of ambulatory function, where gait is impaired on one side of the body, such as amputation, stroke, and hemiplegic CP.

For generally diminished ambulatory function, rule-based algorithms can exploit the fact that gait features typically become less prominent but are not lost. This means that gait remains periodic and features on which rules can be built exist. Applications seeking this category should focus primarily on understanding the unique gait features of the target population. Behboodi et al. [86] used such an approach for gait detection in children with diplegic CP. The authors circumvented the lack of an identifiable heel strike in equinus gait by using angular velocity at the shank, which still shows characteristic peaks, valleys, and zero-crossings.

On the other hand, unilateral loss of ambulatory function typically means that gait features remain on the unimpaired side but are lost on the other. A gait event detection method must thus be extended to additionally capture the irregularities on the impaired side. One approach is presented by Perez et al. [27], who derived a rule-based algorithm from eight other studies, but corrected detection rules that would fail with impaired gait. The authors claim that, whereas normal gait is regular and smooth, the thresholds typically used to detect gait events are tricked by the irregularities found in neurological impaired gait.

Depending on the impairment, assistive devices such as walking frames or orthoses and training devices such as body-weight support systems or exoskeletons can enable or enhance locomotion. The prominence of gait features can then depend on the assistive device. For example, while considering complete SCI patients with functional electrical stimulation (FES), Skelly et al. [13] chose to perform gait event detection using a fuzzy inference system as it can specifically accommodate for the relatively large step-to-step variability observed in FES gait. In Jasiewicz et al. [44], for example, algorithms were significantly under-performing during gaits exhibited when using walking aids. Similarly, with active neuroprostheses such as EES, the stimulation itself can substantially modify the gait pattern [16].

Performing algorithm validations with impaired subjects is essential for advancing clinical applications, but, in doing so, patient safety should not be neglected. All studies in Table 6 besides three, [75,133,135], explicitly state having received ethics approval and obtained informed consent. However, only nine out of 27 mention any safety considerations in their text and only one study has a dedicated section. We recommend that a section on safety always be included in future studies, covering a basic risk analysis and mitigation put in place, and documenting potentially hazardous system failures during experiments. Some example considerations would be preventing skin irritation, ensuring that sensors do not fall off while walking, or verifying that sensors can be securely manipulated by the target population. Such a section will help the community accelerate meaningful development of sensors and algorithms for gait detection and build trust towards their use in clinical applications.

## 4. Conclusions

In the present work, we performed a comprehensive systematic review and provided a broad overview on wearable sensors and methods used in real-time gait analysis. We performed meta-data analysis and identified trends among researchers such as the most sought-after gait events, body segments for IMU placement, and sensor types for ground truth validation of IMU-based gait detection methods. Studies that validated on subjects with impaired gait were then extracted and sensors and methods for clinical applications discussed.

Based on popularity in our findings, we recommend performing gait detection in a subject by using a rule-based method to determine toe off and heel strike. We propose that an IMU is placed on either the foot or shank, and insole pressure sensors are used as ground-truth for validation. When investigating new gait detection methods for clinical use, it is crucial that they are evaluated on their target population and across relevant conditions such as using various walking aids.

One of the limitations of the present review is that the performance of gait detection methods could not be compared quantitatively due to the heterogeneity of the metrics used across the algorithm types. Therefore, we suggest that future studies report performance metrics that would allow benchmarking with respect to comparable methods.

In our review method, we did not consider clinical applications explicitly. However, a subset of the reviewed publications revealed that the algorithm performance can be heavily influenced by gait impairments up to a point where the impairment dictates the algorithm choice. We believe it is a very relevant direction worth further systematic investigation.

Real-time gait detection using wearable sensors provides an unprecedented means to deliver clinical interventions for people with gait impairments. As opposed to traditional gait detection equipment, wearable sensors can inherently be used in an ambulatory setting, and, compared to offline gait analysis, real-time gait detection can be integrated in closed-loop control. The right combination of sensor and gait detection method thus enables the development of assistive devices that have the potential to increase the effectiveness of rehabilitation and improve the lives of people with ambulatory deficits.

## Figures and Tables

**Table 3:** Classification of the most commonly used gait features into intra-stride and inter-stride as well as into temporal, spatial, and spatio-temporal features. The scope of this review is primarily limited to the intra-stride temporal gait features (ISTGFs) highlighted in blue.

Features	Intra-Stride Features	Inter-Stride Features
Temporal	Gait events	
	Gait phases	
	Step duration	Stride duration
	Swing/stance duration	Cadence
Spatial	Step length	Stride length
Spatio-temporal	Joint angles	
	Segment angles, segment positions	
	Joint torques	
	Ground reaction force	
	Centre of pressure	

**Table 4:** Classification of studies based on the type of gait analysis methods used; the number of studies which followed a type of method is listed in the table. Note that a few studies were counted in more than one category when those studies involved more than one method.

Domain	Algorithm	Number of Studies	
Time domain	Rule-based methods	63	92
	Fuzzy inference system (FIS)	4	
	Machine learning (ML)	19	
	Phase portrait (PP)	1	
	Other	5	
Frequency domain	Adaptive oscillator (AO)	4	5
	Spectral analysis	1	
Time-frequency domain	Wavelet transform (WT)	3	4
	Empirical mode decomposition	1	

**Table 5:** Distribution of gait detection techniques for studies that validated on unimpaired subjects. Details on the usage of inertial measurement units (IMU) are presented together with the total number of unimpaired subjects the algorithms types were validated on. ML—Machine learning.

Algorithm	Total Number of Studies	Number of Studies that Used IMU	Number of Studies That Used More than One IMU per Leg	Number of Studies Where the Proposed Method Can Work Independently on Either One of the Legs	Total Number of Unimpaired Subjects	References
Rule-based method	51	38	4	32	485	[7,14,27,37,39,44,46,55,57,59,60,62,68,69,71,72,79,81,82,83,84,85,86,87,88,89,90,91,92,93,94,95,96,97,98,99,100,101,102,103,104,105,106,107,108,109,110,111]
Fuzzy inference system	3	0	0	0	14	[42,73,112]
Hidden Markov model	8	7	3	2	70	[3,6,40,45,64,113,114]
Support vector machine	2	1	1	0	30	[75,115]
Bayesian	2	2	2	2	18	[116,117]
Other ML methods	3	3	2	3	20	[118,119,120]
Phase portrait	1	1	0	1	1	[76]
Lookup table	1	1	0	1	1	[121]
Other time domain methods	4	2	1	1	42	[122,123,124,125]
Adaptive oscillators	4	1	1	0	29	[6,79,83,126]
Wavelet transform	3	3	0	3	61	[80,127,128]

**Table 6:** Algorithm types categorized with respect to the impairment of subjects on which they were validated. Impairments are categorized based on how they were characterized in the respective study. The number of impaired and unimpaired subjects involved in the study suggest the reliability and popularity of the given algorithmic approach for that specific impairment. Note that some studies (such as [63]) are listed more than once in the table depending on whether they employed more than one category of impaired subjects. FIS—Fuzzy inference system, ANFIS—Adaptive neuro fuzzy inference system, HMM—Hidden Markov model.

Impairment	Algorithm Type	Sensor Type	References	Number of Impaired Subjects	Number of Unimpaired Subjects
Parkinson’s disease	Rule-based method	IMU	[55]	16	12
		IMU	[104]	5	15
	Wavelet transform	IMU	[127]	48	40
Osteoarthritis	Support vector machine	IMU + IPS	[75]	14	10
Huntington’s disease	HMM	IMU	[63]	10	0
Cerebral palsy	Rule-based method	IMU	[86]	5	7
		IPS	[92]	3	8
	ANFIS	EMG	[74]	8	0
	HMM	IMU	[113]	10	10
Spinal cord injury	Rule-based method	IMU	[44]	14	26
	FIS	IPS	[13]	3	0
Elderly	Spectral analysis	IMU	[129]	92	0
	HMM	IMU	[63]	10	0
Amputee	Rule-based method	IMU	[7]	1	8
		IMU	[37]	1	9
		IMU + IPS	[69]	3	5
		IPS	[110]	1	1
Stroke	Rule-based method	IMU	[130]	2	0
		IMU + IPS	[131]	1	0
		IMU	[132]	1	0
		IMU	[90]	4	10
		IMU	[133]	1	0
		IMU	[104]	4	15
		IMU	[134]	6	0
		IMU	[97]	10	22
		IMU	[135]	2	0
		IMU	[27]	1	1
	HMM	IMU	[63]	10	0
Unspecified Hemiplegia/ Hemiparesis	Rule-based method	IMU	[14]	10	10
	HMM	IMU	[3]	10	10

## Data Availability

Not applicable.

## Appendix A. Background of Wavelet Transform Method

One way to extract gait cadence is through frequency spectrum analysis. The two prominent peaks in the frequency spectrum analysis of gait signals (e.g., angular velocity of the foot) correspond to stride frequency and step frequency (first and second harmonics). Cadence can be evaluated from one of the two peaks. However, to extract cadence in real-time, we need to perform a Fourier transform (FT) over a window of data streamed continuously, the so-called short-time Fourier transform.

STFT suffers from poor resolution in the time-frequency domain [11]. To improve the resolution in one domain, the resolution in the other domain must be compromised. For instance, to get better resolution of frequency content, we need to provide it with a larger (time) window of data, thereby reducing the resolution in the time domain, resulting in output cadence evaluated over a larger interval (see Figure A1). Hence, STFT can be considered as a suitable technique for long duration, steady-state walking (quasi-periodic with no abrupt changes), but not for fast motion transitions [11].

The constraint of the resolution trade-off can be overcome with a wavelet transform (WT). Much like an FT, a WT also decomposes the signal based on a set of basis functions. While these basis functions are sinusoids in the case of FTs, the basis functions in the case of WTs are wavelets. The difference is that, while sinusoids only differ in their frequencies, wavelets are localized both in the time and the frequency domain. Therefore, unlike STFT, which uses windows of fixed size in time domain, a WT uses windows of varying size in the time-frequency domain, increasing the time resolution with high frequency signals and vice versa [136,137].

There are two variants of discrete wavelet transform (DWT). The first variant, where ‘discrete’ implies discretized time-frequency domain while otherwise being identical to continuous wavelet transform (CWT), is described in [137] and implemented in [11]. Han et al. [11] used this version of DWT to extract step frequency/cadence and reports that WT performs better than STFT in estimating step frequency when fast motion changes occur. The second variant, which is filter bank-based, is described in [136] and implemented in [138]. Klingbeil et al. [138] carried out step detection using this version of DWT with a Daubechies wavelet. Here, the signal is split into the so-called detail levels and then reconstructed with detail levels spread across 0.8 Hz to 3.2 Hz (assuming most of the gait signal is in this range), followed by step detection using thresholds. The MATLAB implementations of both version of DWT are available, but the former is called by the name *cwt* (because of its resemblance to CWT), while the latter is called by the name *dwt*. Note that, although Refs. [11,138] carried out the study in real-time, these studies were limited to step detection and step frequency, but not ISTGF detection. Our own implementation of wavelet transform for gait detection, based on *cwt*, is shown in Figure A2.

One limitation of WT is the high computational complexity required for such an implementation, which we also noticed in our own implementation presented in Figure A2. Since WTs involve comparing the signal to wavelets, the transform at the beginning and end of the signal are less reliable because the wavelet cannot be overlapped completely with the extreme ends of the signal. This leads to the so-called cone of influence, a region outside of which the result of wavelet transform is no longer reliable. This is usually of low significance in offline analysis since the entire signal is available for analysis at once; however, in real-time analysis, the cone of influence becomes more important. This is because what we are interested in for every iteration is the transform of the latest sample of data, which is precisely where the reliability is poor.
